# Solubilisation of Phosphate and Micronutrients by *Trichoderma harzianum* and Its Relationship with the Promotion of Tomato Plant Growth

**DOI:** 10.1371/journal.pone.0130081

**Published:** 2015-06-25

**Authors:** Rui-Xia Li, Feng Cai, Guan Pang, Qi-Rong Shen, Rong Li, Wei Chen

**Affiliations:** 1 Jiangsu Key Lab and Engineering Center for Solid Organic Waste Utilization, Nanjing Agricultural University, Nanjing, China; 2 National Engineering Research Center for Organic-based Fertilizers, Nanjing, China; 3 Jiangsu Collaborative Innovation Center for Solid Organic Waste Resource Utilization, Nanjing Agricultural University, Nanjing, China; Agroecological Institute, CHINA

## Abstract

*Trichoderma harzianum* strain SQR-T037 is a biocontrol agent that has been shown to enhance the uptake of nutrients (macro- and microelements) by plants in fields. The objective of this study was to investigate the contribution of SQR-T037 to P and microelement (Fe, Mn, Cu and Zn) nutrition in tomato plants grown in soil and in hydroponic conditions. Inoculation with SQR-T037 significantly improved the biomass and nutrient uptake of tomato seedlings grown in a nutrient-limiting soil. So we investigated the capability of SQR-T037 to solubilise sparingly soluble minerals *in vitro* via four known mechanisms: acidification by organic acids, chelation by siderophores, redox by ferric reductase and hydrolysis by phytase. SQR-T037 was able to solubilise phytate, Fe_2_O_3_, CuO, and metallic Zn but not Ca_3_(PO_4_)_2_ or MnO_2_. Organic acids, including lactic acid, citric acid, tartaric acid and succinic acid, were detected by HPLC and LC/MS in two *Trichoderma* cultures. Additionally, we inoculated tomato seedlings with SQR-T037 using a hydroponic system with specific nutrient deficiencies (i.e., nutrient solutions deficient in P, Fe, Cu or Zn and supplemented with their corresponding solid minerals) to better study the effects of *Trichoderma* inoculation on plant growth and nutrition. Inoculated seedlings grown in Cu-deficient hydroponic conditions exhibited increases in dry plant biomass (92%) and Cu uptake (42%) relative to control plants. However, we did not observe a significant effect on seedling biomass in plants grown in the Fe- and Zn-deficient hydroponic conditions; by contrast, the biomass decreased by 82% in the P-deficient hydroponic condition. Thus, we demonstrated that *Trichoderma* SQR-T037 competed for P (phytate) and Zn with tomato seedlings by suppressing root development, releasing phytase and/or chelating minerals. The results of this study suggest that the induction of increased or suppressed plant growth occurs through the direct effect of *T*. *harzianum *on root development, in combination with indirect mechanisms, such as mineral solubilisation (including solubilisation via acidification, redox, chelation and hydrolysis).

## Introduction

Nutrient deficiencies give rise to significant agronomic problems despite the large extent to which fertiliser are used in modern agriculture [1]. For example, despite the large quantity of P in soils, this element is a major plant growth-limiting nutrient because most of it is easily fixed in the form of insoluble phosphates [2]. Other elements, such as Fe, Mn, Cu and Zn, which are involved in a number of physiological and metabolic processes, are also not always active in soils. Moreover, the increase in cultivation intensity due to the increasing demand for higher yields has led to faster depletion of microelements from soils, resulting in micronutrient-deficiencies in many intensively exploited soils [3]. Fe, Mn, Cu and Zn are required for balanced plant nutrition, and their deficiencies have great impacts on yields and the quality of agricultural products. Thus, methods that increase the bioavailability and uptake of these important nutrients are necessary and of particular scientific interest. The biological activities of microorganisms (e.g., *Trichoderma* spp.) in soil mediate to a large degree the solubility, and thus the availability, of nutrient minerals at the root surface [4]. Soil microorganisms have been reported to alter soil pH and to modify the equilibrium of many chemical and biochemical reactions [1].

*Trichoderma* spp. are effective biocontrol agents for a number of soil-borne pathogens and are also known for their ability to promote plant growth [5,6]. However, to date, the majority of the research on *Trichoderma* has focused on biocontrol, while the use of these microbes to enhance plant nutrition, and especially micronutrient nutrition, has not been extensively studied. Although there is some evidence that this important biocontrol agent can increase the bioavailability of insoluble or sparingly soluble elements (e.g., P and Fe, Mn, Cu and Zn), previous research on this topic has yielded inconsistent results [3,7,8]. For example, a study by Yedidia et al. [9] showed that the concentrations of P, Fe, Mn, Cu, Zn and Na in *Trichoderma*-inoculated cucumber roots significantly increased, whereas de Santiago et al. [10] reported that *Trichoderma asperellum* competes for Cu, Mn and Zn with plants and decreases the concentrations of these elements in plants. Thus, while it has been shown that *Trichoderma* plays a role in nutrient solubilisation and uptake, the detailed effects of *Trichoderma* inoculation remain to be determined.

Several mechanisms by which *Trichoderma* may influence plant development have been proposed, such as the production of phytohormones, the solubilisation of sparingly soluble minerals, the induction of systemic resistance in the host plant, a reduction in pollutant toxicity (organic or heavy metal), and the regulation of rhizospheric microflora [3,11,12,13,14]. Altomare et al. [7] did not detect the release of organic acids by *T*. *harzianum* strain T22, while Adams et al. [8] reported that metal chelation via organic acids and proteins were the main mechanisms by which *T*. *harzianum* T22 increased metal desorption. Thus, the mechanisms by which this process occurs are unclear and remain topics of ongoing investigation. *T*. *harzianum* strain SQR-T037 is a plant growth-promoting fungus that is sold commercially (Patent Application No. 200910233576.1) in China as a biocontrol agent. In our previous work [12], we demonstrated that *T*. *harzianum* SQR-T037 released an auxin-like phytohormone (harzianolide) that significantly increased the total root length and the numbers of root tips of tomato plants 1.5- to 2.6-fold. However, it is unclear whether *T*. *harzianum* SQR-T037 promotes plant growth directly by enhancing root development or via the production of metabolites that lead to the solubilisation of minerals.

The objectives of the present work were the following: 1) to determine whether *T*. *harzianum* SQR-T037 is able to improve the uptake of several nutrients (P, Fe Mn, Cu, and Zn) by plants, and 2) to determine the mechanisms by which *T*. *harzianum* regulates plant growth and solubilises nutrients. The results of this study should explain at least some of the plant growth-promoting effects of *T*. *harzianum* and provide new insights into its interactions with plants.

## Materials and Methods

### Microbial strain

*T*. *harzianum* strain SQR-T037 (CGMCC accession No. 3308, China General Microbiology Culture Collection Center) was provided by the National Engineering Research Center for Organic-Based Fertilizers in Nanjing, China. It was routinely cultured on potato dextrose agar (PDA) at 28°C and was preserved on slants at 4°C [12].

### Pot experiments

To analyse the effect of SQR-T037 inoculation on plant nutrient uptake in nutrient-limiting soil, pot experiments were conducted under greenhouse conditions (24–28°C, 60–80% relative humidity, light: dark = 14 h: 10 h). Specifically, 15-day-old tomato seedlings (*Lycopersicon esculentum* cv. Suhong 2003) were each inoculated with 5 ml of SQR-T037 spore suspensions (SS, 10^6^ colony form units [CFU] per ml) after planting, using uninoculated seedlings as a control (CK). Each pot (diameter = 10 cm, height = 9.5 cm) contained 400 g of soil and one tomato seedling. The soil was a clay loam with a pH of 8.1, 13.2 g kg^-1^ of organic matter, 7.9 mg kg^-1^ of ammonium-N, 25.3 mg kg^-1^ of nitrate-N, 2.0 mg kg^-1^ of available P, 134.7 mg kg^-1^ of available K, 1.1 mg kg^-1^ of available Fe, 0.6 mg kg^-1^ of available Mn, 0.1 mg kg^-1^ of available Cu, and 0.1 mg kg^-1^ of available Zn. Five replicates of each treatment were randomly arranged, and irrigation and loosening were done when needed. After 4 weeks, chlorophyll was measured in triplicate on the last two completely expanded leaves using a Minolta SPAD-502 (Minolta Camera Co, Ltd., Osaka, Japan). Then, all of the seedlings were carefully removed from the pots, and data regarding seedling biomasses and nutrient concentrations were collected as described below. For each treatment, rhizospheric soil samples were collected from the sampled plant roots as described by Hervás et al. [15]. Each soil sample was mixed thoroughly and divided into 2 parts for dilution plating (stored at 4°C) and nutrient analysis (air-dried at room temperature).

### Solubilisation of minerals in SQR-T037 liquid culture

For solubilisation experiments, a modified version of the methods described by Altomare et al. [7] was used. The minerals (20 mg) used in the present study were Ca_3_(PO_4_)_2_, Fe_2_O_3_, MnO_2_, CuO and metallic Zn which were added in powder form to 50 ml of sucrose-yeast extract (SY) medium separately. Three replicate flasks were sampled each day for 9 days to determine the pH values and soluble mineral contents in the culture medium. The mycelium was removed by filtration (0.45 μm), and the P, Fe, Mn, Cu and Zn contents in the filtrates were determined using an inductively coupled plasma optical emission spectrometer (710 ICP-OES, Agilent Technologies, California, USA). Uninoculated media in flasks that were processed in the same way were used as the control.

### Analysis of organic acids in SQR-T037 culture filtrates

Organic acids were reported to contribute to the solubilisation of minerals through acidification of the microenvironment near the roots and the sequestration of minerals [3]. In this experiment, *T*. *harzianum* SQR-T037 was incubated in SY medium (50 ml) [7] or glucose broth (50 ml) [8] at 28°C. After 9 days, the culture filtrates from the two media were both analysed for the presence of 16 organic acids (propanedioic, ferulic, lactic, succinic, oxalic, fumaric, malic, tartaric, citric, p- hydroxybenzoic, vanillic, phthalic, salicylic, cinnamic, mandelic and syringic acids) via comparison with a set of standards (Sigma Chemical Company, USA) using an Agilent 1200 semi-preparative HPLC (Agilent Technologies, Santa Clara, USA) equipped with an Agilent ZORBAX Eclipse XDB-C18 analytical column (4.6×250 mm, 5 mm). Briefly, the culture filtrates from the SY medium (hereafter termed Sample 1) were extracted with ethyl acetate to remove low polar components, and then the water phase was analysed by HPLC; the culture filtrates from the glucose broth (hereafter termed Sample 2) were analysed directly. The mobile phase consisted of 5 mM H_2_SO_4_ (0.4 ml min^-1^) and was detected with a single-wavelength UV detector at 210 nm. The suspected peaks were further identified with an Agilent LC/MS 6410B (Agilent Technologies, Santa Clara, USA) using the positive ion mode. In addition, the titratable acid of the culture filtrates from the glucose broth were measured using 0.01 mol L^-1^ NaOH each day for 9 days.

### Analysis of phytase activity, siderophore production and ferric reductase release due to SQR-T037

Although acidification is an important mechanism of mineral solubilisation, it is not the only possible one. Chelation and reduction by other metabolites, such as siderophores and ferric reductases, may also play a role [3,16]. Soil organic P accounts for 40–80% of total P, and among all the forms of organic P, phytate is usually expected to be the most abundant in soils [17]. Thus, the activity of phytase (which transforms phytate-P into plant-available P) in SQR-T037 cultures was estimated by measuring the soluble P in cultures with ICP-OES assay (710 ICP-OES, Agilent Technologies, California, USA). Petri dishes (9 cm in diameter) were prepared with calcium phytate medium (CPM, containing 0.5% calcium phytate, 2% dextrose, 0.5% NH_4_NO_3_, 0.05% KCl, 0.05% MgSO_4_, 0.01% FeSO_4_, 0.001% MnSO_4_, and 0.1% Triton X-100 per litre), and the phytase activity in the corresponding liquid medium was measured each day for 9 days. Siderophores were detected using the modified chrome azurol S (CAS) assay, as described by Machuca and Milagres [18]. Czapek-Dox liquid cultures were used to measure ferric reductase release, and the absorbances at 562 nm were measured each day. Fungal biomass, the production of siderophores and ferric reductase release were measured in triplicate daily for 9 days.

### Hydroponic experiments

To better characterise the growth response of tomato plants to mineral solubilisation by SQR-T037, experiments were performed in a hydroponic system in a controlled environment (24°C, 80% relative humidity, and a circadian cycle of 14 h:10 h light:dark). The treatments were the same as those for the pot experiments (SS and CK). Uniform 15-day-old tomato seedlings were transplanted into 250-ml hydroponic containers containing 100 ml of nutrient solution. Each treatment group consisted of at least 10 hydroponic containers containing 2 seedlings each. After 3 weeks, the seedlings were sampled and measured as described above for the pot experiments. Data regarding the root growth of seedlings was collected using a root scanner (Epson perfection V700 Photo, SEIKO EPSON corp., Japan). Based on the results of the mineral solubilisation experiments above, the *Trichoderma*-soluble elements were chosen for further testing. For example, if the solubilisation experiments showed that SQR-T037 could solubilise Fe_2_O_3_, we would culture tomato seedlings in Fe-deficient nutrient solution (using the Yamazaki nutrient solution for reference, pH 6.5) supplemented with solid Fe_2_O_3_ (50 mg each container).

### Analysis of plant and soil samples

At the end of the experiments, plant samples (shoots and roots) were dried at 70°C for 5 days to measure dry weights. Dried plant materials were ground and digested with concentrated HNO_3_-H_2_O_2_ using the methods described by Yedidia et al. [9]. The digests were used to determine P, K, Fe, Cu, Mn and Zn content using an ICP-OES (710 ICP-OES, Agilent Technologies, California, USA). The N content of plant samples was analysed with a Vario EL elemental analyser (Elementar Analysensysteme GmbH, Hanau, Germany). Soil nutrient properties, including soil organic matter, available P and available K content, were determined as described by Shen et al. [19]. Specifically, soil organic matter was determined by the dichromate oxidation. Soil available P and K were extracted with sodium bicarbonate and ammonium acetate, respectively, and then determined by the molybdenum-blue method and a flame photometry, respectively. Soil nitrate- and ammonia-N were analysed using a continuous-flow analyser (AutoAnalyser 3, Bran+Luebbe GmbH, Germany). The quantities of available Fe, Cu, Mn, and Zn in soils were determined using an ICP-OES assay (710 ICP-OES, Agilent Technologies, California, USA), in which the soil samples were digested by diethylene triamine pentaacetic acid. The colonised population of *T*. *harzianum* SQR-T037 in tomato rhizospheres was obtained by dilution plating using *Trichoderma*-selective medium [20]. The data were expressed as the number of CFU per gram of dry soil.

### Statistical analysis

The means and standard deviations of the data were calculated and statistically examined by ANOVA and using Duncan’s multiple range test. SPSS software was used for these analyses (SPSS, Inc., Chicago, IL, USA). The significance level was set at *P* < 0.05 unless otherwise stated. All of the experiments were conducted at least twice.

## Results

### Growth response to SQR-T037 inoculation in soil

**Fig 1:**
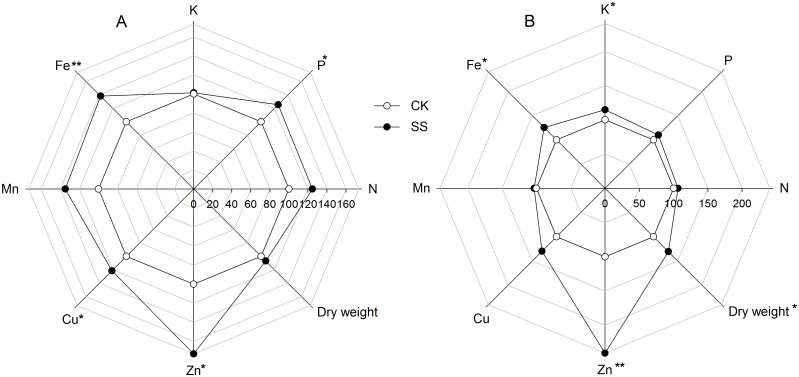
Plant biomass, as indicated by dry weight and macro- and micro-element concentrations of tomato roots (A) and shoots (B) grown in nutrient-limiting soil. SS, seedlings inoculated with *Trichoderma* SQR-T037 spore suspensions; CK, uninoculated controls. Mean percentages (control = 100%) were calculated from 5 sampled tomato seedlings. Absolute values for the controls (100%) were as follows: (A) dry weight, 0.143 g plant^-1^; N, 22.75 g kg^-1^; P, 1.03 g kg^-1^; K, 25.03 g kg^-1^; Fe, 44.83 mg kg^-1^; Mn, 16.69 mg kg^-1^; Cu, 1.18 mg kg^-1^; and Zn, 10.12 mg kg^-1^; (B) dry weight, 0.815 g plant^-1^; N, 26.00 g kg^-1^; P, 1.41 g kg^-1^; K, 39.78 g kg^-1^; Fe, 34.03 mg kg^-1^; Mn, 3.43 mg kg^-1^; Cu, 0.56 mg kg^-1^; and Zn, 6.62 mg kg^-1^. Values of *P < 0.05 and **P < 0.01 (ANOVA) were considered to represent statistically significant differences.

The population of *Trichoderma* in tomato rhizospheric soil was 3.0×10^4^ CFU per gram of dry soil, as measured by dilution plating. The pot experiments showed that inoculation with *T*. *harzianum* SQR-T037 significantly (*P* < 0.05) affected the biomass and nutrient uptake of tomato seedlings. The seedling dry weight increased by 31%, and K, Fe and Zn uptake increased by 15–40% in the tomato shoots; the uptake of P, Fe, Cu and Zn by the roots was also higher (21–73%) than in the uninoculated control (Fig 1). In contrast, no significant effect of *Trichoderma* inoculation was found on N or Mn uptake. The available nutrient contents in the soils at the end of the pot experiments are given in S1 Table.

### Solubilisation of sparing soluble minerals by SQR-T037

**Fig 2:**
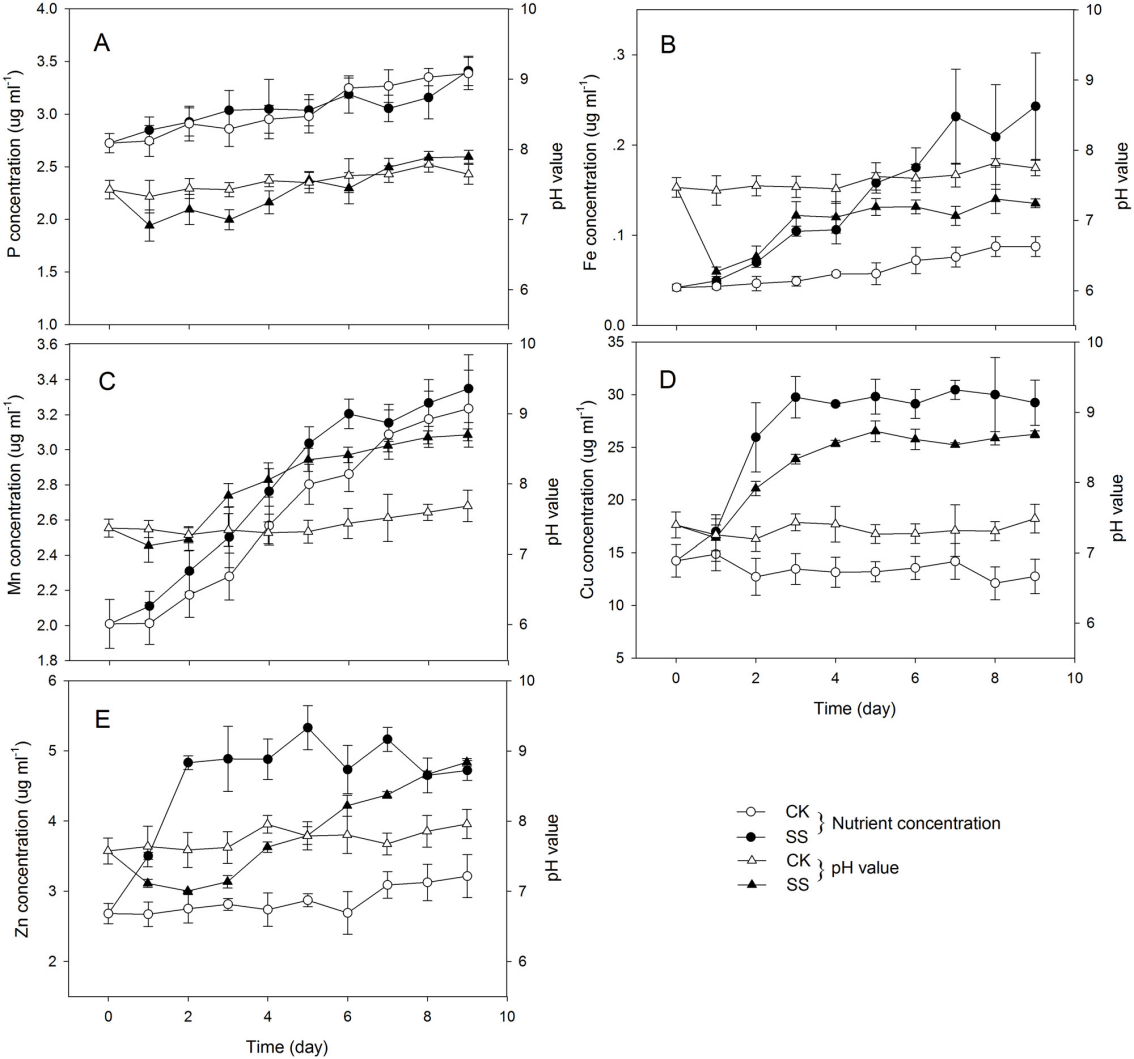
Concentrations of soluble P (A), Fe (B), Mn (C), Cu (D) and Zn (E) in *Trichoderma* SQR-T037 cultures in sucrose-yeast exact medium supplied with Ca_3_(PO_4_)_2_, Fe_2_O_3_, MnO_2_, CuO and metallic Zn, respectively. SS, cultures inoculated with *Trichoderma* SQR-T037 spore suspensions; CK, uninoculated controls. Measurements of soluble nutrients and medium pH values were performed daily from the 0th to the 9th day. Error bars indicate the standard deviations of 3 replicates.

Experiments were conducted in duplicate to investigate the ability of *Trichoderma* cell-free culture filtrates to solubilise several minerals. Solubilisation of Fe_2_O_3_, CuO and metallic Zn was demonstrated by an increase in the concentrations of the corresponding soluble nutrients. The Fe concentration in liquid culture following *Trichoderma* inoculation rose from 0.04 to 0.24 μg ml^-1^, the Cu concentration rose from 14.2 to 29.2 μg ml^-1^, and the Zn concentration rose from 2.7 to 4.7μg ml^-1^, while the concentrations of these minerals in the control samples remained almost constant over time (Fig 2B, 2D and 2E). However, solubilisation of Ca_3_(PO_4_)_2_ or MnO_2_ was not detected, since no significant difference in the concentrations of the corresponding soluble elements between the SS treatment and the control (CK) was observed (Fig 2A and 2C).

### Production of organic acids in SQR-T037 culture filtrates

**Fig 3:**
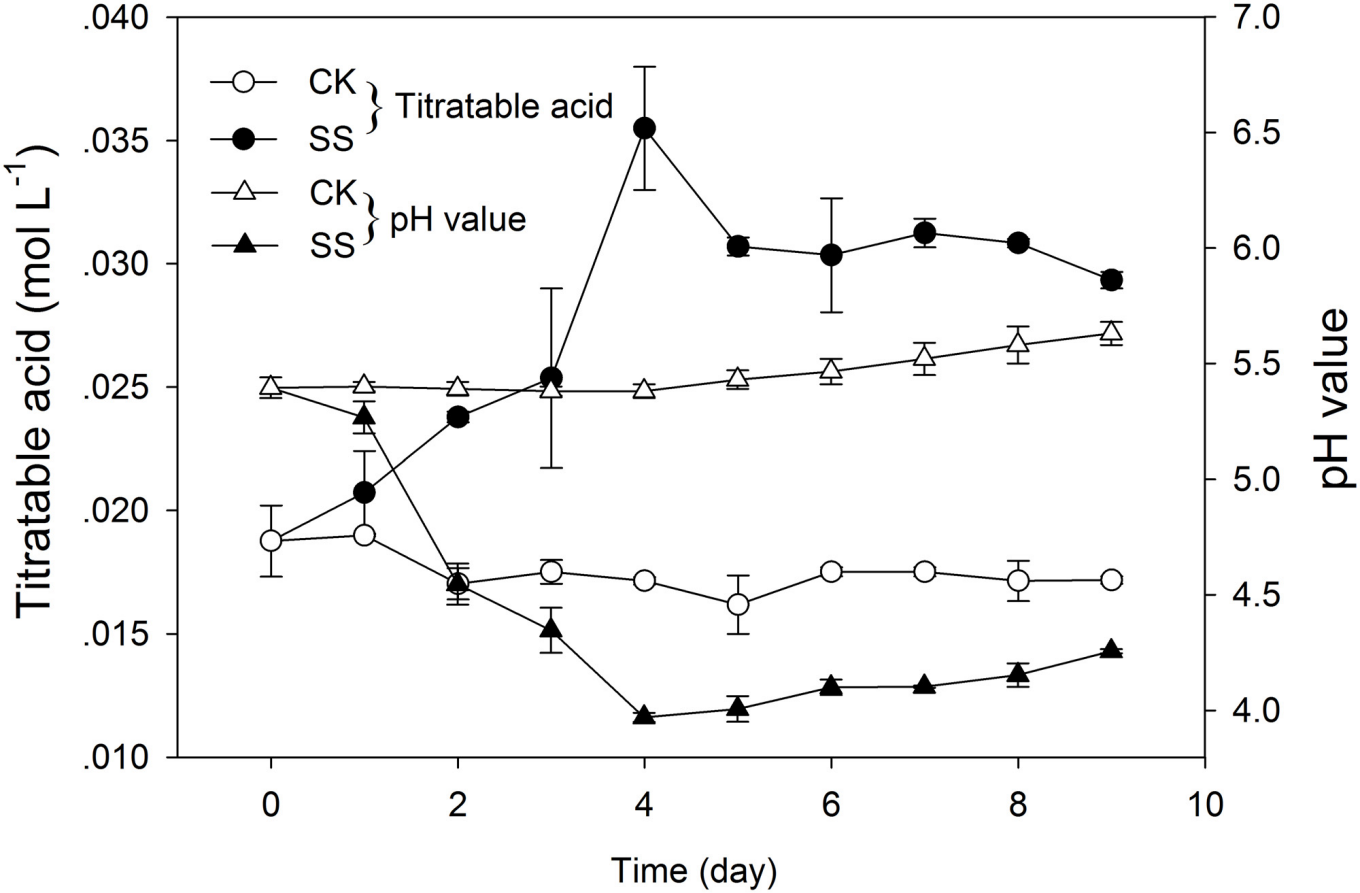
Assays measuring titratable acids and pH in glucose broth over time. Samples containing 3 ml of the uninoculated control (CK) and *Trichoderma*-inoculated cultures (SS) were measured using 0.01 mol L^-1^ NaOH each day from days 0 to 9. Error bars indicate the standard deviations of 3 replicates.

Four organic acids were identified in SQR-T037 culture filtrates based on matches between their retention times and those of the standards (S1 and S2 Figs) and based on comparisons of their molecular weights (Table 1). The production of lactic acid and citric acid in Sample 1 was confirmed by the presence of the corresponding product ions ([2M + H]^+^ = 181 and 385 a.m.u., respectively) and of their adducts with sodium ([2M + Na]^+^ = 203 and 407 a.m.u., respectively). Similarly, tartaric acid and succinic acid (Esi+ m/z 173 [M + Na]^+^, 189 [M + K]^+^ and 207 [M + K + H_2_O]^+^ for tartaric acid; 259 [2M + Na]^+^ and 275 [2M + K]^+^ for succinic acid) were found in Sample 2. However, the other 12 organic acids that were examined were not detected in either of the two culture filtrates. Titratable acid analyses showed an increase in NaOH consumption (from 0.018 mol L^-1^ to 0.035 mol L^-1^) and a decrease in pH values (from pH 5.4 to pH 4.3) in SQR-T037 culture filtrates over time (Fig 3).

**Table 1:** LC/MS detection of organic acids obtained from two culture filtrates of *Trichoderma* SQR-T037.

Samples^a^	Organic acids	Precursor ion (m/z)	Product ions (m/z)
Sample 1	Lactic acid	90	181, 203
	Citric acid	192	385, 407
Sample 2	Tartaric acid	150	173, 189, 207
	Succinic acid	118	259, 275

^a^Sample 1 and Sample 2 were collected from sucrose-yeast extract medium and glucose broth, respectively. *Trichoderma* were allowed to grow in the media at 28°C for 9 days.

### Quantification of phytase activity, siderophore production and ferric reductase release due to SQR-T037

**Fig 4:**
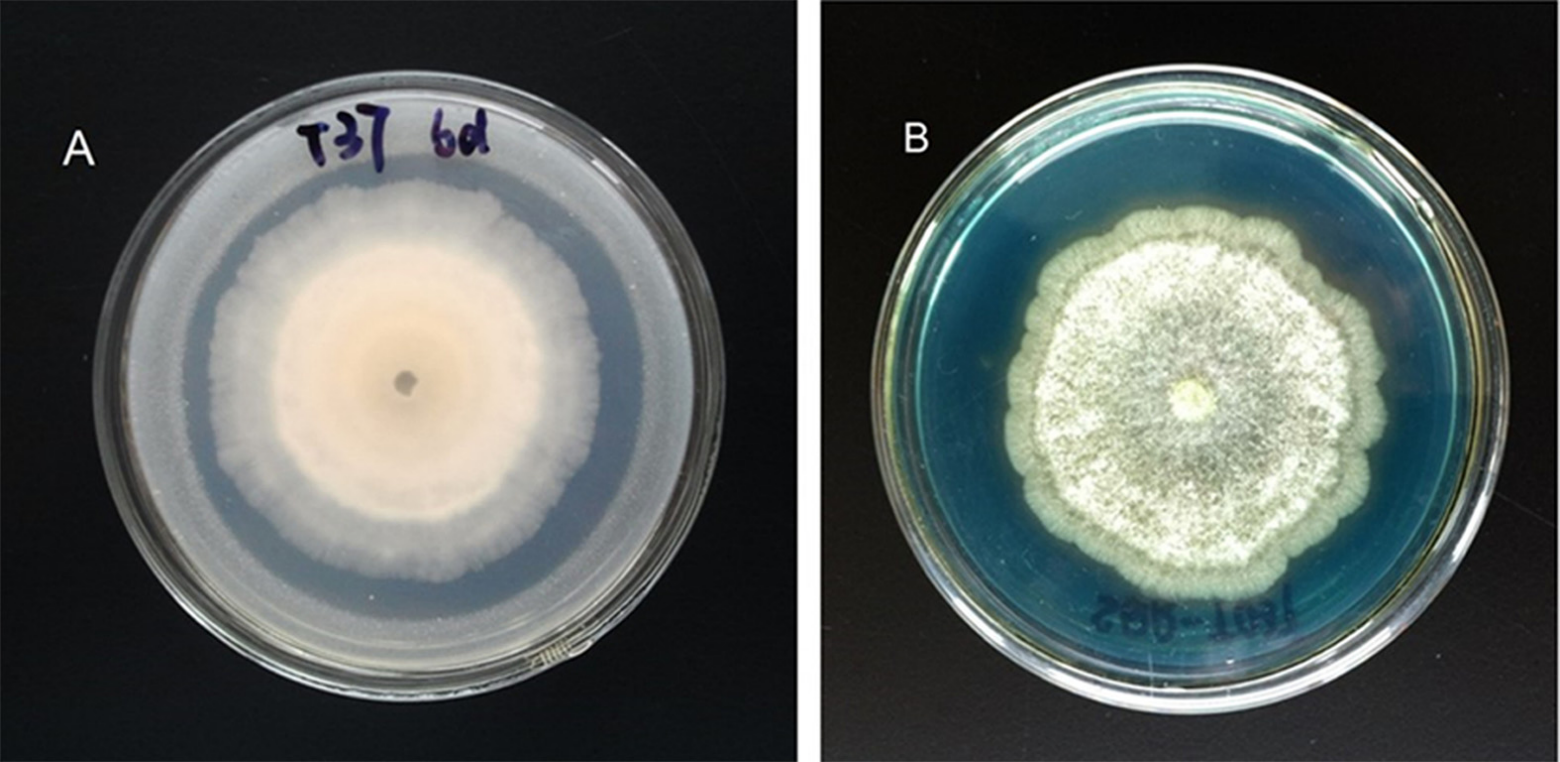
Formation of halos on calcium phytate medium (A) and modified chrome azurol S medium (B) by *Trichoderma* strain SQR-T037. Images were taken on the 6th day post inoculation.

**Fig 5:**
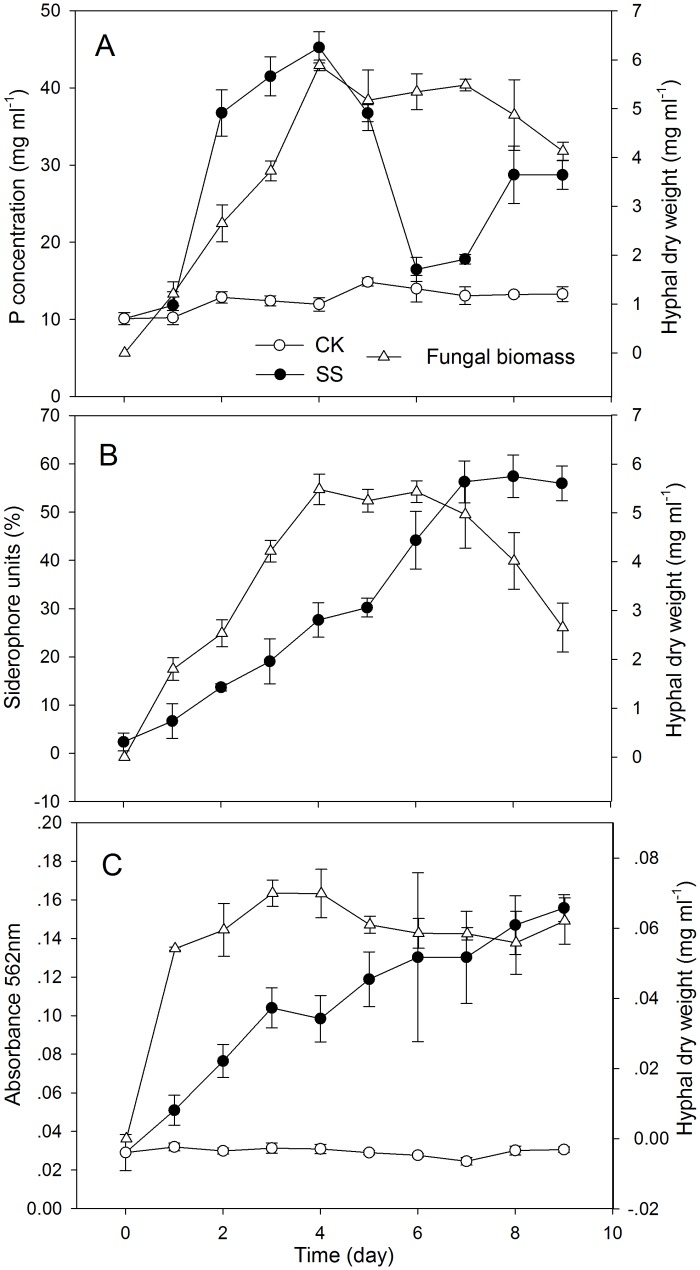
Quantification of phytase activity (A), siderophore production (B) and ferric reductase release (C) by *Trichoderma* SQR-T037. Phytase activity was expressed as the P concentration in liquid calcium phytate medium. Siderophore production was expressed as the percentage of the siderophore unit relative to the control. Ferric reductase release was expressed as the absorbance at 562 nm. SS, cultures inoculated with *Trichoderma* SQR-T037 spore suspensions; CK, uninoculated controls. Measurements of enzymatic activity, siderophore production and fungal biomass (hyphal dry weight) were made daily from days 0 to 9. Error bars indicate the standard deviations of 3 replicates.

*Trichoderma* strain SQR-T037 produced a clear halo around its colonies in CPM and CAS agar (Fig 4). Phytate solubilisation, Fe chelation and Fe reduction were estimated quantitatively after incubation from the 0th to the 9th day (Fig 5). The soluble P in *Trichoderma* culture filtrates (SS) rapidly increased with hyphal growth during the first 4 days and reached its peak value (45.2 mg ml^-1^) on the 4th day. Then, phytase hydrolysis declined, while the fungal biomass remained constant. Soluble P began to increase again from the 7th day, while the fungal biomass decreased with time (Fig 5A). Fig 5B shows that the production of siderophores in the SS cultures increased up to 56% over time compared with the uninoculated controls (CK). The absorption spectrophotometry analysis of ferric reductase release showed that the absorbance of the SS cultures increased over time (from 0.03 up to 0.16), indicating that the presence of ferric reductase gradually grew over time, whereas the absorbance in the CK cultures remained almost unchanged (Fig 5C).

### Growth response to SQR-T037 inoculation in nutrient-deficient hydroponic culture conditions

**Fig 6:**
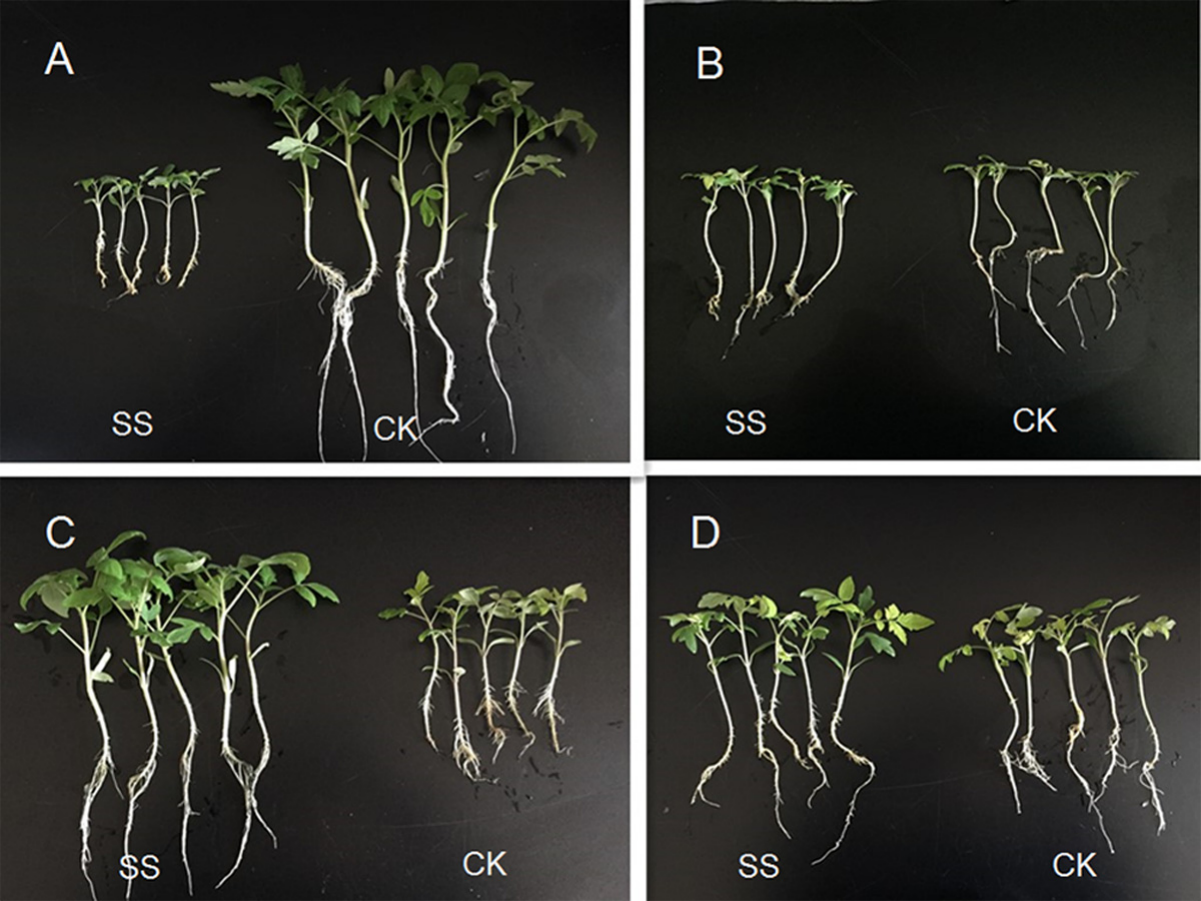
Tomato seedlings grown in P-, Fe-, Cu- or Zn-deficient nutrient solutions [supplied with phytate (A), Fe_2_O_3_ (B), CuO (C) and metallic Zn (D), respectively] 3 weeks after sowing. SS, seedlings inoculated with *Trichoderma* SQR-T037 spore suspensions; CK, uninoculated controls.

The effect of mineral solubilisation by *Trichoderma* SQR-T037 on plant growth was initially evaluated by measuring the chlorophyll content (shown in SPAD units), dry weight, and macro- and microelement concentrations of plants (Table 2; Fig 6). Next, we initiated a P-deficient hydroponic experiment (HE-P), in which tomato seedlings were grown in a P-deficient nutrient solution supplemented with a solid phytate supply. In these conditions, we observed a significant decrease in plant biomass (82%, dry weight), K concentration (46%) and Mn concentration (30%) in SQR-T037-inoculated plants (SS) compared to uninoculated control plants (CK) (*P* < 0.01). However, the SQR-T037-inoculated plants exhibited higher concentrations of N (24%), Fe (83%) and Zn (109%) than did the uninoculated controls. In Fe-deficient hydroponic conditions (HE-Fe, supplied with solid Fe_2_O_3_), the leaves of SQR-T037-inoculated seedlings (SS) exhibited less chlorosis than did the leaves of control plants (CK). Correspondingly, the SPAD readings and Fe concentrations of tomato plants were increased by 24% and 146% relative to control plants, respectively. However, this increased Fe uptake did not enhance plant growth, as measured by plant dry weight. A prominent effect of SQR-T037 inoculation (SS) was found on the growth of tomato seedlings grown in Cu-deficient hydroponic conditions (HE-Cu, supplied with solid CuO). Significant increases of 58%, 92%, 57%, 42% and 65% were observed in the SPAD readings, the dry weight, and the K, Cu and Zn concentrations of SQR-T037-inoculated seedlings, respectively. Inoculation with *Trichoderma* SQR-T037 (SS) in Zn-deficient hydroponic conditions (HE-Zn, supplied with solid Zn) did not significantly affect the dry weights of tomato seedlings and decreased the SPAD readings and the concentrations of Fe and Zn in these tomato plants (*P* < 0.05).

**Table 2:** Effect of *Trichoderma* inoculation on tomato plant growth and mineral concentrations in hydroponic experiments.

Treatments^a^		SPAD readings	Dry weight (mg plant^-1^)	N (g kg^-1^)	P (g kg^-1^)	K (g kg^-1^)	Fe (mg kg^-1^)	Mn (mg kg^-1^)	Cu (mg kg^-1^)	Zn (mg kg^-1^)	*Trichoderma*Population (10^4^ CFU ml^-1^)
HE-P	CK	31.81±2.33	93.63±12.82	30.20±0.11	3.81±0.07	90.88±3.22	175.84±4.31	160.63±6.86	6.38±0.98	111.60±1.18	-
	SS	34.55±3.05	16.57±1.73**	37.32±0.51**	4.88±0.83	48.69±1.56**	322.18±14.73**	113.01±4.53**	8.11±3.23	233.28±20.51*	18.50±2.50
HE-Fe	CK	21.19±2.07	19.73±3.49	41.55±0.98	11.09±0.33	63.90±2.39	374.23±8.33	437.68±49.72	43.89±6.25	143.00±24.64	-
	SS	26.20±2.98*	22.67±3.95	41.39±0.90	11.03±0.42	61.81±2.86	918.75±21.55**	179.53±4.70*	15.93±1.52*	316.53±74.68	7.25±1.25
HE-Cu	CK	16.80±2.66	32.40±6.84	39.30±0.84	8.87±0.32	70.15±3.18	126.52±5.11	152.15±15.77	139.91±8.49	95.32±15.96	-
	SS	26.50±3.33**	62.36±7.41**	42.62±0.69	8.99±0.81	110.21±4.85**	58.86±28.01	126.72±0.70	199.20±10.49*	157.74±3.63*	4.17±1.28
HE-Zn	CK	20.07±1.99	35.48±4.61	39.43±0.28	9.02±0.19	69.78±5.28	348.93±10.46	176.71±4.87	18.19±2.73	2318.89±55.62	-
	SS	15.11±2.70*	36.92±5.62	40.73±0.07*	8.60±0.07	65.26±5.49	149.41±25.74**	195.93±16.28	17.85±0.79	1266.48±97.84**	5.50±1.25

^a^Tomato plants inoculated with *Trichoderma* spore suspensions in roots (SS) or non-inoculated controls (CK). HE represents hydroponic experiments, e.g., He-P indicates that tomato seedlings were grown in a P-deficient nutrient solution (using the Yamazaki nutrient solution for reference, pH 6.5) supplemented with solid phytate (50 mg per container); seedlings were also grown in conditions similarly deficient in Fe (Fe_2_O_3_), Cu (CuO) and Zn (metallic Zn). Data were collected 21 days post inoculation. Quantification of the *Trichoderma* population in the different nutrient-deficient solutions was performed using the standard 10-fold dilution plating method to quantify colony-forming units (CFU). The data are expressed as the mean values ± standard deviations (n = 3 for nutrients assay; n = 10 for other indexes). Statically significant differences were obtained from a one-way ANOVA, and the significance levels between treatments were set at

*P < 0.05 and **P < 0.01.

**Fig 7:**
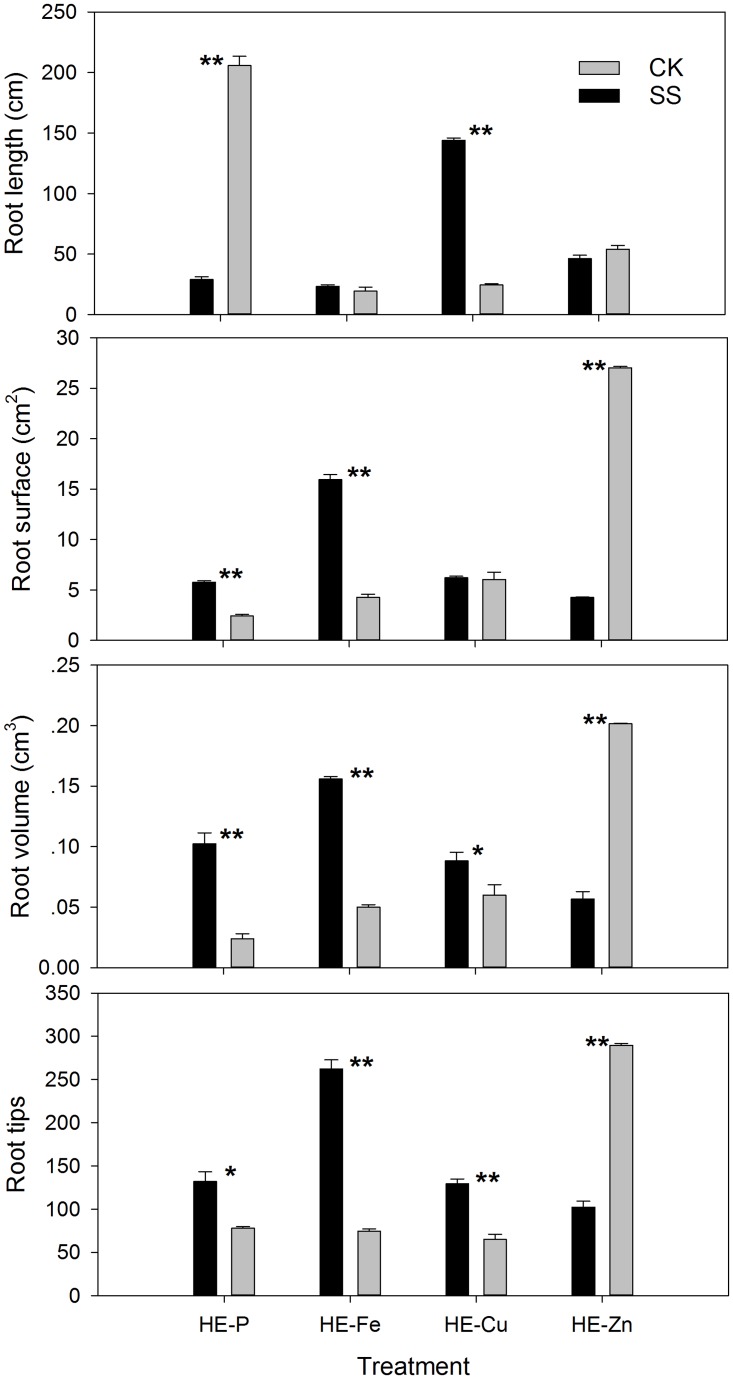
Root growth in tomato seedlings grown in P-, Fe-, Cu- or Zn-deficient nutrient solutions [supplied with phytate (A), Fe_2_O_3_ (B), CuO (C) and metallic Zn (D), respectively] 3 weeks after sowing. SS, seedlings inoculated with *Trichoderma* SQR-T037 spore suspensions; CK, uninoculated controls. Error bars indicate the standard deviations for 5 biological replicates. Statistically significant difference between treatments were set at *P < 0.05 and **P < 0.01 (ANOVA).

*Trichoderma* SQR-T037 colonised tomato roots well under these experimental conditions. This was especially evident in the HE-P condition, in which *Trichoderma* population reached 10^5^ CFU ml^-1^. Moreover, root analysis suggested a mechanism by which *Trichoderma* SQR-T037 affected plant growth and nutrient uptake (Fig 7): in the HE-P condition, the tomato root surface area, the root volume and the numbers of root tips were increased by SQR-T037 inoculation, whereas limited growth in root lengths occurred. By contrast, nearly the opposite phenomenon of root development was found in the HE-Zn condition, in which the root surface area, the root volume and the numbers of root tips of SQR-T037-inoculated plants (SS) decreased by 16%, 28% and 35%, respectively, compared with the control (CK). All parameters of root development following SS treatment in the HE-Fe and the HE-Cu conditions were improved compared to the control (CK), although no significant difference in the root length in the HE-Fe condition and the root surface area in the HE-Cu condition was observed between the SS-treated and control plants.

## Discussion

In the hydroponic experiments, however, improved plant growth following *Trichoderma* inoculation was found only in the Cu-deficient condition (Table 2), whereas the hydroponic conditions with Fe or Zn deficiency caused no significant difference in plant growth (*P* < 0.05) between SS treatment and control conditions. Additionally, we observed an 82% decrease in plant biomass following SS treatment in P-deficient hydroponic conditions. Thus, the results presented here confirm for the first time that *T*. *harzianum* may use different mechanisms when faced with different nutrient deficiencies, resulting in different influences on plant growth.

The ability of *T*. *harzianum* to solubilise sparingly soluble minerals (including Fe_2_O_3_, MnO_2_, CuO, Zn and rock P) *in vitro* was demonstrated by Altomare et al. [7]. In our study, the solubilisation of Ca_3_(PO_4_)_2_ or MnO_2_ was not detected; no significant difference in the mineral concentrations between the *Trichoderma*-inoculated cultures and the controls was detected (Fig 2). However, analyses of P in the CPM medium revealed measurable concentrations of soluble P following SS treatment that were significantly different from the control concentrations, suggesting that phytase released by *Trichoderma* played an important role in solubilising organic P (i.e., phytate). Furthermore, solubilisation of Fe_2_O_3_, CuO and metallic Zn were detected under our experimental conditions. On the basis of these observations, the hypothesis that the use of *Trichoderma* SQR-T037 improved nutrient uptake by plants and thus promoted plant growth was supported. Additionally, the results of pot experiments with nutrient-limiting soil supported the hypothesis that increased plant growth and nutrient uptake (P, Fe, Cu and Zn) occurred in *Trichoderma*-inoculated seedlings (Fig 1).

Improved plant growth following inoculation with *T*. *harzianum* SQR-T037 has previously been demonstrated in tomatoes under greenhouse and field conditions [12,21]. However, it was not possible to conclude that the mineral solubilisation by *Trichoderma* was primarily responsible for promoting plant growth because several other mechanisms are involved in *Trichoderma*-plant interactions. Using a hydroponic system to induce specific nutrient deficiencies, we separated the effects of mineral solubilisation and of direct root stimulation by *Trichoderma* from other mechanisms by which *Trichoderma* affects plant growth (e.g., regulation of rhizospheric microflora). In Cu-deficient hydroponic conditions, the improvements in plant growth and nutrient concentrations (K, Cu and Zn) that were observed may be directly related to a general beneficial effect of *T*. *harzianum* inoculation on the root system, as indicated in Fig 7. Increases in root lengths, root volumes and in the numbers of root tips may have enabled the roots to maintain better contact with the minerals examined in this study; these findings are of great significance for nutrient uptake when nutrients are scarce [9]. Additionally, improvements in plant growth may partly result from enhanced Cu availability; this hypothesis was supported by data on CuO solubilisation that we obtained (Fig 2D) and by the correspondingly higher level of Cu (Table 2) found in the inoculated seedlings in the HE-Cu condition. This enhanced Cu availability could in part be the result of chelation by *Trichoderma* but was not due to acidification by organic acids because the pH increased during these solubilisation experiments.

Inoculation with *Trichoderma* SQR-T037 increased the Fe concentration and SPAD values in the tomato seedlings grown in the HE- Fe condition, thus revealing a positive effect of this strain on Fe nutrition but without a corresponding effect on plant biomass (Table 2). Fe must be in reduced form [Fe(II)] to be taken up by plants, while it is found in the soil primarily as insoluble oxyhydroxide polymers or as Fe(III) chelates, especially at alkaline pH. Thus, for roots to take up Fe, Fe(III) oxides must be solubilised beforehand [4]. Most fungi excrete Fe-specific chelators, siderophores, such as coprogen, coprogen B, and ferricrocin [22], to mobilise this metal in response to low Fe availability in the environment [16]. In addition to siderophores, other microbial products, such as organic acids and ferric reductase, may exhibit acidification and chelation properties toward Fe [3]. Here, we showed that ferric reductase and organic acids, including lactic acid, citric acid, tartaric acid and succinic acid, were produced in two different media, demonstrating the involvement of redox and acidification in this process. However, the organic acids examined in this study could not be the major contributors to Fe_2_O_3_ solubilisation because the pH of the medium stayed invariant during days 3 to 9 when the concentrations of Fe continued to increase (Fig 2B). Therefore, *T*. *harzianum* SQR-T037 was able to solubilise Fe_2_O_3_ by multiple mechanisms that involved chelation, reduction of Fe(III) and acidification (Figs 2B, 4B, 5B and 5C).

Phytate remains the most abundant source of organic P in soils due to its stability [16,23]. The halo and quantification of P in the CPM culture showed that *T*. *harzianum* SQR-T037 exhibited high phytase activity, as evidenced by substantial hydrolysation of phytate (Figs 4A and 5A). However, an apparent inhibition of plant growth was found in *Trichoderma*-inoculated seedlings in the HE-P condition (Fig 6A; Table 2). Additionally, the Zn concentration in *Trichoderma*-inoculated seedlings in our HE-Zn experimental condition decreased by 45% relative to control plants (Table 2). de Santiago et al. [10] reported that *Trichoderma* strain T34 significantly decreased the concentration and total amount of Cu, Mn, and Zn in the aerial parts of wheat plants in ferrihydrite-enriched calcareous medium. Thus, we suspected that the adverse effect of SQR-T037 on the Zn nutritional status of plants and plant growth in the P-deficient hydroponic condition was due to competition for nutrients between microorganisms and plants under conditions of restricted availability. Generally, a considerable portion of the P in soil is used by microorganisms to build their cells, and these organisms often exhibit a higher P uptake efficiency than do plant roots. The P incorporated by microorganisms will become available to plants only after the death and lysis of microbial cells [24]. This may also explain why the soluble P content in the CPM cultures decreased during the 4th to the 6th day and then increased again after the 7th day (Fig 5A). Because the solubilisation of metallic Zn could not be attributed to reduction, we hypothesised that it may instead be due to acidification via the production of organic acids; this hypothesis was supported by the detection of lower pH values compared to control conditions, as shown in Fig 2E.

Inoculation with *T*. *harzianum* resulted in increased Zn uptake and root growth in cucumber plants and crack willow saplings, as reported by Yedidia et al. [9] and Adams et al. [8], respectively. In contrast to these previous results, in the present study, lower Zn nutrition detected in inoculated seedlings was accompanied by suppressed root development in the HE-Zn condition (Table 2; Fig 7). These results, together with those from the HE-Cu and HE-Fe conditions, suggested a common role for *Trichoderma* in regulating the plant root system. As suggested by some authors, *Trichoderma* strains could be defined as plant symbiont opportunistic organisms that are able to colonise plant roots by mechanisms similar to those used by mycorrhizal fungi to stimulate plant growth, possibly via the production or control of phytohormones [5,6,12].

Taken together, these results suggest that the induction of increased or suppressed plant growth occurs through the direct effect of *T*. *harzianum* on root development, in combination with indirect mechanisms, such as mineral solubilisation (including solubilisation via acidification, redox, chelation and hydrolysis). The mechanisms by which *Trichoderma* regulates plant growth and mineral solubilisation partly depend on the absence of a given element. For example, under the experimental conditions described in this work, *T*. *harzianum* SQR-T037 competed for P (phytate) and Zn with tomato seedlings by suppressing root development, and root length, in particular. By contrast, *T*. *harzianum* SQR-T037 promoted plant growth via a combination of directly facilitating root development and increasing nutrient uptake and dissolution (i.e., acidification, redox and chelation for Fe, and most likely chelation for Cu). And these results were also supported by the data of pot experiments except Zn, which may due to the different growing conditions between soil and hydroponic system for *Trichoderma*. While these data are promising, further research is still required to examine the specific effects of *T*. *harzianum* on plant growth via each mechanism. Additionally, the ability to solubilise insoluble nutrients to promote plant growth among *Trichoderma* species is probably strain-specific.

## Supporting Information

S1 Fig Chromatogram of sample 1 (blue) and the standard sample of citric acid (red) and lactic acid (green). The mobile phase for HPLC analysis was 5 mM H_2_SO_4_ (0.4 ml min^-1^) and was detected with a single-wavelength UV detector at 210 nm.
(TIF)

S2 Fig Chromatogram of sample 2 (blue) and the standard sample of succinic acid (red) and tataric acid (green). The mobile phase for HPLC analysis was 5 mM H_2_SO_4_ (0.4 ml min^-1^) and was detected with a single-wavelength UV detector at 210 nm.
(TIF)

S1 Table Effects of *Trichoderma* inoculation on the soil available nutrients in the pot experiments. Samples were collected from the plant roots sampled for each treatment at the end of pot experiments. Tomato seedlings were allowed to grow in pots for 4 weeks. The data are expressed as the mean values ± standard deviations (n = 5). Statistically significant differences were determined by a one-way ANOVA, and the significance levels between treatments were set at *P < 0.05 and **P < 0.01.
(DOCX)
