# Supplementary figures and images for: Solubilisation of Phosphate and Micronutrients by Trichoderma harzianum and Its Relationship with the Promotion of Tomato Plant Growth
Source: PLoS One. 2015 Jun 25;10(6):e0130081. doi: 10.1371/journal.pone.0130081 (PMC4482446; doi:10.1371/journal.pone.0130081)

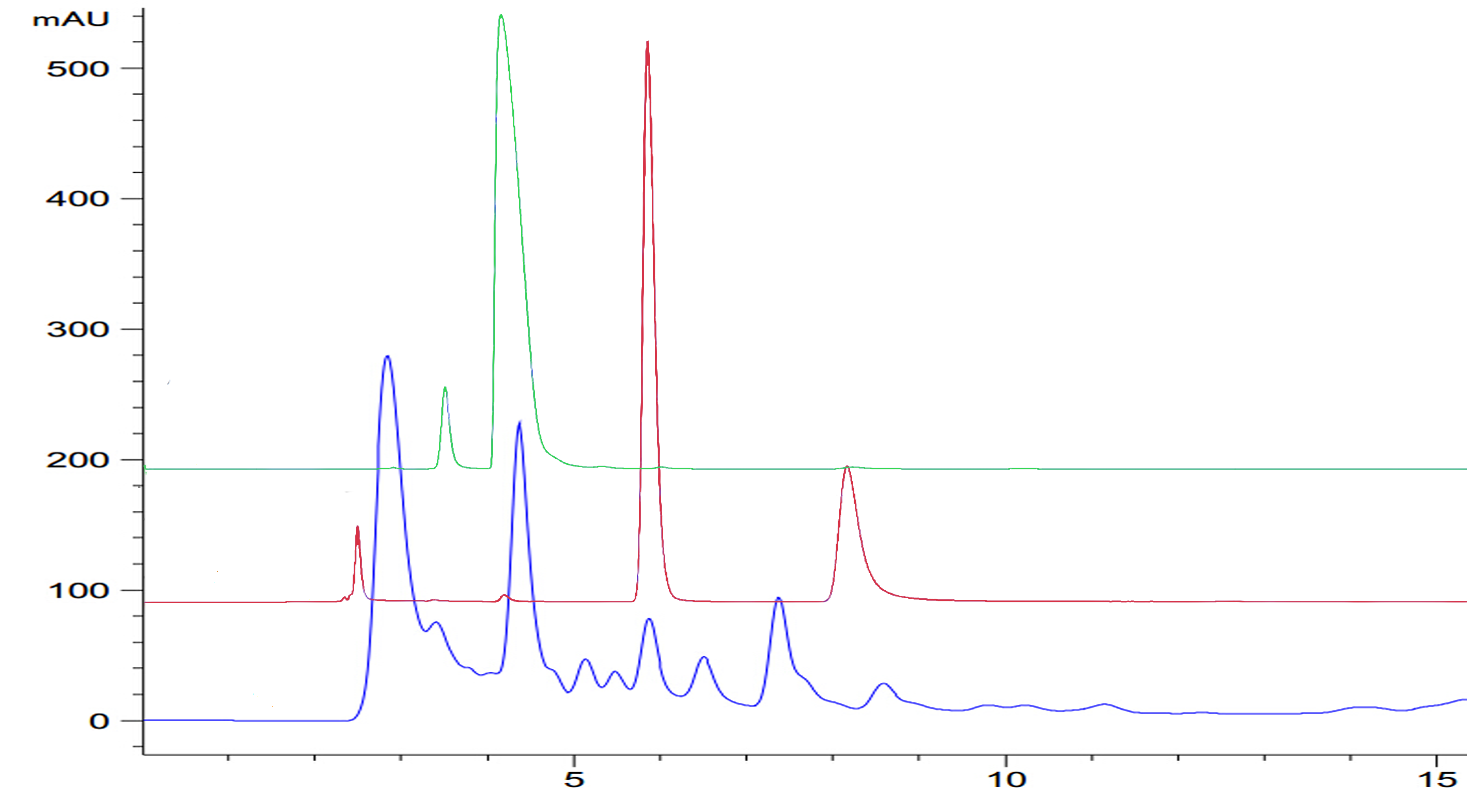

Supplement: S1 Fig — The mobile phase for HPLC analysis was 5 mM H2SO4 (0.4 ml min-1) and was detected with a single-wavelength UV detector at 210 nm. (TIF) [file pone.0130081.s001.tif]

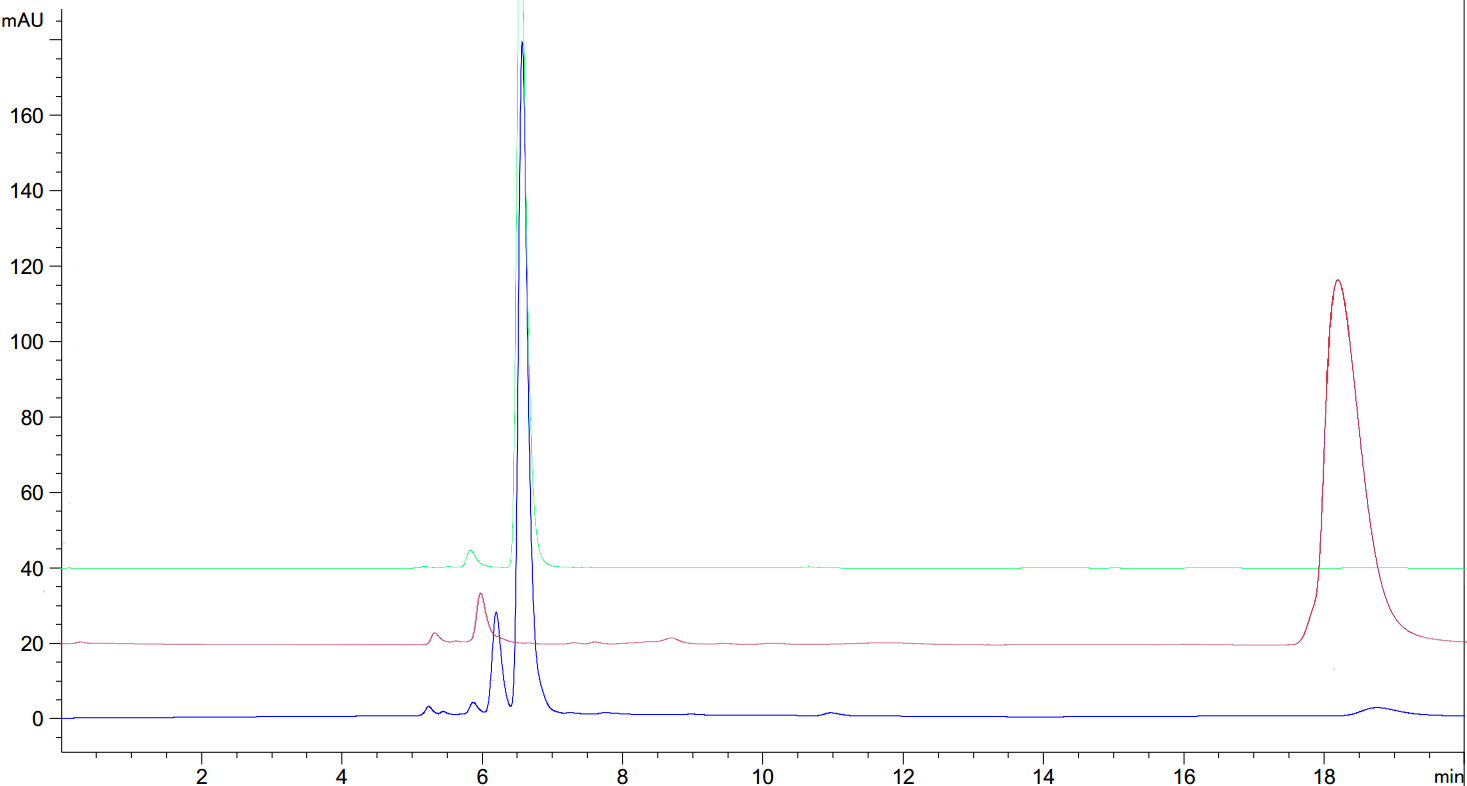

Supplement: S2 Fig — The mobile phase for HPLC analysis was 5 mM H2SO4 (0.4 ml min-1) and was detected with a single-wavelength UV detector at 210 nm. (TIF) [file pone.0130081.s002.tif]
