# Supplementary material for: Solubilisation of Phosphate and Micronutrients by Trichoderma harzianum and Its Relationship with the Promotion of Tomato Plant Growth
Source: PLoS One. 2015 Jun 25;10(6):e0130081. doi: 10.1371/journal.pone.0130081 (PMC4482446; doi:10.1371/journal.pone.0130081)
Supplement: S1 Table — Samples were collected from the plant roots sampled for each treatment at the end of pot experiments. Tomato seedlings were allowed to grow in pots for 4 weeks. The data are expressed as the mean values ± standard deviations (n = 5). Statistically significant differences were determined by a one-way ANOVA, and the significance levels between treatments were set at *P < 0.05 and **P < 0.01. (DOCX) [file pone.0130081.s003.docx]

**Table S1** Effects of *Trichoderma* inoculation on the soil available nutrients in the pot experiments.

| Treatments^a^ | Ammonia-N  (mg kg^-1^) | Nitrate-N  (mg kg^-1^) | Available P  (mg kg^-1^) | Available K  (mg kg^-1^) | Available Fe  (mg kg^-1^) | Available Mn  (mg kg^-1^) | Available Cu  (mg kg^-1^) | Available Zn  (mg kg^-1^) |
| --- | --- | --- | --- | --- | --- | --- | --- | --- |
| CK | 10.73±0.50 | 23.14±3.13 | 1.39±0.82 | 285.29±3.67 | 15.9±0.83 | 18.98±1.86 | 1.55±0.18 | 0.43±0.06 |
| SS | 12.24±1.64 | 26.40±0.28 | 1.13±0.21 | 303.74±1.62 | 22.79±0.93^*^ | 26.21±1.19^*^ | 1.94±0.05^*^ | 0.47±0.01 |

^a^Samples were collected from the plant roots sampled for each treatment at the end of pot experiments. Tomato seedlings were allowed to grow in pots for 4 weeks. The data are expressed as the mean values ± standard deviations (n = 5). Statistically significant differences were determined by a one-way ANOVA, and the significance levels between treatments were set at ^*^*P* < 0.05 and ^**^*P* < 0.01.
